# Collaborative training of regulators as an approach for strengthening regulatory systems in LMICs: experiences of the WHO and Swissmedic

**DOI:** 10.3389/fmed.2023.1173291

**Published:** 2023-05-18

**Authors:** Razieh Ostad Ali Dehaghi, Alireza Khadem Broojerdi, Lodovico Paganini, Hiiti B. Sillo

**Affiliations:** ^1^Regulation and Safety Unit, World Health Organization, Geneva, Switzerland; ^2^Swiss Agency for Therapeutic Products (Swissmedic), Bern, Switzerland

**Keywords:** collaborative training, regulatory systems strengthening, NRA, RSS, WHO, Swissmedic, training, regulator

## Abstract

**Introduction:**

Training opportunities for health product regulators are among the critical aspects in the strengthening of regulatory systems across the world. The need for training is reasonably higher among the National Regulatory Agencies (NRAs) in the Low- and Middle-Income countries (LMICs) which are faced with many regulatory challenges mostly rooted in the low availability of resources. The current study aimed at evaluating the suitability, impacts, and challenges related to the training of regulators from LMICs offered by the Swissmedic in collaboration with the World Health Organization (WHO).

**Methodology:**

An exploratory case study design using a qualitative approach was adopted to collect data from a total of 17 NRAs in different WHO regions using in-depth interviews and qualitative questionnaires.

**Results:**

The participation of the trainees in the training was revealed to be motivated by the need to apply the obtained knowledge in addressing various challenges within their NRAs. Many lessons covering all key areas of health products regulation were reported by the trainees, whereby most of the lessons were already being implemented within their respective NRAs. However, challenges related to human, financial, and infrastructural resources were highlighted to hinder the ongoing efforts in putting the learned aspects into practice. Additionally, areas in which further regulatory assistance and suggestions for improving the training activities were pointed out.

**Conclusion:**

The highlighted gains from the WHO-Swissmedic collaborative training program call for other agencies and organizations to join hands in offering much-needed support towards addressing critical challenges facing the regulatory sector in the LMICs.

## 1. Introduction

Training refers to a systematic way of fostering the gaining of knowledge, skills, and attitudes *via* different forms of instruction, demonstration, and practical exposure (1, 2). Since human resources is an extremely valuable part of any organization, staff training is associated with a broad range of benefits to the organization. These include increased productivity, competitiveness, sustainability, and general profitability through the provision of better products or services (1, 2). Moreover, training gives chance to trainees to attain higher competencies, and increased motivation towards fulfilling the required tasks, among other benefits (1, 3). Providing ongoing training and re-training opportunities to employees should therefore be regarded as among the core responsibilities of accountable employers.

There is a critical need for training among the health products regulators in Low- and Middle-Income Countries (LMICs). This is mainly contributed by limited opportunities for formal training in regulatory sciences, high turnover rates among staff, and fast advancements in knowledge and technology related to health products (3–6).

To address the challenges around the limited competencies of regulators across the world, the WHO drafted a competency-based framework to guide the training activities for regulators (7). Moreover, several other competency models for regulatory professionals have been put forward by other groups (5). However, despite the frameworks and other curricula, the shortage of other resources such as trainers, training materials, finance, and training infrastructure has continued to impair the training of regulators, particularly among the LMICs (5, 6).

To address some of those challenges, the Regulatory Systems Strengthening (RSS) team of the World Health Organization (WHO) and the Swiss Institute for Therapeutic Products (Swissmedic) collaboratively organized regulatory training activities aimed at regulators from the LMICs. The training was developed in accordance with the WHO’s support to the implementation of the National Regulatory Agencies’ (NRA) Institutional Development Plans (IDP) *via* strengthening the capacity of the participating regulators through experience sharing and knowledge exchange.

The NRAs participating in the training are invited by the WHO based on a set of pre-selected criteria, including their regulatory preparedness. The invited NRAs are mandated to select the staff to attend the respective training based on their prevailing needs for capacity building, among other factors. A total of 30–35 trainees are usually invited per training round, whereby they are trained *via* oral presentations, group discussions and presentations, case studies, and giving of reading materials. To enhance interactions between the trainers and the trainees, the aspects of having questions and answers sessions, supervised discussions and exercises are built into the programme.

The training is delivered by the sharing of knowledge and skills on up-to-date methods and procedures for processes in areas of Quality Management System (QMS), Registration and Marketing Authorization (MA), Market Surveillance and Control (MC) and Vigilance (VL) in accordance with the WHO and other international standards and good practices. The capacity-building component of the training is intended to not only directly benefit the participants on an individual basis; rather it is meant to generate a deeper impact on the performance of their respective NRAs in the various aspects covered during the training. This study, therefore, aimed at evaluating the suitability, impacts, and challenges associated with the collaborative offering of training to regulators as an approach to strengthening regulatory systems in LMICs.

## 2. Methodology

An exploratory case study design using a qualitative approach was adopted to explore the factors influencing trainees’ selection and their motivation, lessons learnt, their implementation, existing challenges and suggestions from the trainees and their immediate supervisors, as well as their trainers (8, 9). The study was conducted among regulators from different NRAs in the LMICs who have attended at least one of the previous regulatory training rounds between 2018 and 2021. Moreover, trainers from the Swissmedic and trainees’ immediate supervisors from their respective NRAs were involved.

A total of 17 NRAs from the countries shown in Table 1 were purposefully sampled from 45 NRAs whose regulators had taken part in the training at the onset of the study. Moreover, purposeful sampling was employed to recruit individual trainees from across different years/rounds of attending the training, regardless of their roles/functions within the NRAs. The same sampling procedure was used to recruit 3 trainers from the Swissmedic based on their levels of experience in delivering the training. Furthermore, referral sampling was used to recruit 7 immediate supervisors of the trainees who took part in this study.

**Table 1:** Summary of the studied NRAs with their respective number of participants, WHO region and World Bank economic status.

WHO region	Country/NRA (study participants)	World Bank’s economic status
African Region (AFR)	Rwanda (2), Sierra Leone (1), South Sudan (2)	Low income
	Kenya (2), Nigeria (2), South Africa (2), Tanzania (3)	Lower-middle income
Eastern Mediterranean Region (EMR)	Egypt (3), Pakistan (1)	Lower-middle income
European Region (EUR)	Albania (2)	Lower-middle income
	Armenia (1)	Upper-middle income
South-East Asian Region (SEAR)	Sri Lanka (3)	Lower-middle income
Western Pacific Region (WPR)	Malaysia (2), Mongolia (2), Papua New Guinea (2), Philippines (3)	Lower-middle income
	Fiji (1)	Upper-middle income

In-depth small groups- (2–3 participants) or one-to-one interviews were conducted between January – July 2022. A semi-structured interview guide was used to explore the perceptions, experiences and suggestions of the trainees and trainers 6 months to 4 years after taking part in the previous training rounds. The interview guide contained open-ended questions designed to probe aspects before, during and after attending the training (Supplementary material). The suitability of the interview guides for training participants was tested *via* a pilot study involving the first 2 NRAs. Following the pilot study, further modifications were made to increase clarity, enhance flexibility in the order of the questions and ensure that the allocated time was adequate.

All interviews were conducted by the same interviewer using video calls on an online based platform (Zoom Video Communications, California, United States). Each interview was conducted in the English language and lasted for a total of 45–60 min. A saturation of shared information as revealed by the recurrence of similar aspects/themes was realized after 13 interviews with the trainees, however, 4 more were conducted to arrive at a total of 17 interviews (34 trainees).

Responses from the trainees’ immediate supervisors were obtained using a qualitative questionnaire survey entailing open-ended questions targeting long-form written answers (Supplementary material). To ensure that the questionnaire probe on the meant aspects, the questions were drafted by an independent moderator before being appraised by all authors to ascertain if all aspects intended to be surveyed are present. Furthermore, the responses from the first three questionnaires were reviewed by a pair of authors and an independent moderator to establish if they were truly within the context of each question. In the same way, the rigor of the questionnaire was evaluated based on the consistency of the responses from those participants. Following this step, responses from four other immediate supervisors were collected.

Data analysis was carried out by inductive thematic analysis as previously described by Braun and Clarke (10). This was based on the large nature of the dataset, the scarcity of literature on similar aspects, and the need for higher flexibility in the identification, analysis and reporting of the possible themes and patterns (10, 11). To familiarise with the data, the video records were watched and listened to once before they were subsequently transcribed verbatim. We conducted a manual transcription as oppose to using a “qualitative data analysis software” in order to enhance understanding of respondents feedback. In addition, this process was adopted to focus more attention on the meaning and depth of the data, as well as allowing for more flexible, team-oriented, and transparent coding and themes allocation processes.

Following the transcription process, six of the generated transcripts were randomly selected and validated by a pair of authors who did not participate in the generation of the transcripts.

Thereafter, all transcripts were further read and re-read by a team of four authors to identify the meanings, senses and initial patterns in the interviews.

Secondly, initial codes were generated from a systematic appraisal of the entire data set aimed at identifying many distinct patterns possible while capturing both semantic and latent features of the data. Corresponding data extracts were thereafter matched and collated to each of the identified codes whereby, some extracts were coded more than once. Moreover, some new codes identified during the coding and analysis process were added to the initial codes.

Searching for themes was done by allocation of related codes and associated data extracts into possible themes. Through further careful evaluation of the existing relationships, candidate sub-themes and themes were generated. A further rearrangement of the candidate themes and sub-themes was done while paying close attention to their internal homogeneity and external heterogeneity. This was achieved by team-oriented reviewing of coded data extracts as well as entire data set levels. This process was associated with dividing, merging, and discarding the candidate themes based on their ultimate validity.

Furthermore, defining and naming the themes was done through proper identification of the essence and scope of the data captured in each theme. This was followed by the generation of the accompanying detailed narratives under each theme. Ultimately, the writing of the final report was conducted by a generation of a complete description of the data to provide a concise, coherent, and logical account of the information available in the analysed data.

Each study participant was issued a detailed informed consent and took part voluntarily. To ensure the confidentiality of the information, the NRAs’ and participants’ identities were concealed upon the first transcription, whereby codified identifications were issued.

## 3. Results

### 3.1. Factors influencing trainees’ selection and motivation

Several factors were reported to influence the nominations of the NRA staff to take part in the training. These included their current role(s), having a particular leadership position, educational background, and level of experience were among the stated bases for their nominations. Particularly, trainees denoted the need for acquiring more knowledge to be capable of addressing the required Corrective and Preventive Actions (CAPAs) from the previous WHO benchmarking activity in their NRA. It was anticipated that attending the training will sharpen their knowledge and skills, hence ensuring the successful addressing of the CAPAs.

Moreover, other trainees pointed out specific areas in which they anticipated an in-depth understanding of their handling by the Swissmedic. These included the registration procedures, reliance systems, pharmacovigilance of Covid-19 vaccines, Quality Management Systems (QMS) and use of Information Management systems (IMS) in regulatory activities. In addition, trainees reported looking forward to the creation of new partnerships towards having more opportunities for regulatory collaboration and an exchange of ideas and/or experiences.

### 3.2. Key lessons from the training and putting learnt aspects into practice

Lessons of diverse nature were highlighted by the trainees. The lessons cut across areas of regulatory collaboration, pharmacovigilance (PV), organization of regulatory systems, effective evaluation of medicines, and the use of IMS. Moreover, lessons around the concept of reliance, modalities for seeking technical assistance, as well as identifying and adopting best practices from other NRAs were shared. For example, some trainees reported shifting their focus from only generating PV reports to ensuring the presence of functional PV systems.

Furthermore, the trainees reported on lessons on the importance of having well-structured and operational systems, good organization, delegation and oversight of regulatory functions, as well as good political support and legal frameworks. Other areas included a better understanding of QMS, risk-based principles, effective training of regulators, digitalization of regulatory procedures, careful planning for available resources and strict measures to keep agreed timelines.

*“… we were privileged to know most of the things, especially with the QMS in the Swissmedic, … they are not necessarily interested with certifications but with ensuring that the system is in place, is running and is also in use … The main thing is for people to know what they are supposed to do and continually implement it …, we should move our focus from ‘certification, certification’ (Trainee 9, NRA 4).*

The trainees reported implementing various aspects learnt from the training within their NRAs. The implementation of these changes was aimed at shortening processing time, optimizing the use of the available resources, improving access to medicines, and avoiding unnecessary regulatory procedures.

Implemented changes in regulatory practices involved the establishment of online registration systems, the creation of specific windows for the submission of applications, and the dropping of laboratory analyses for Market Authorization (MA) renewals. Also reported were the creation or expansion of technical committees, the establishment of reliance and other collaborative systems, as well as the application of various risk minimization measures.

*“… seeing an SRA like Swissmedic doing a reliance approach encouraged us to proceed in this aspect too. This helps us to keep our resources for products which really need deeper and closer evaluations…” (Trainee 3, NRA 2).*

Other trainees reported on the creation and modification of various regulatory documents of various natures. In some NRAs this went along with the development of documents related to the overall legal framework.

*“Before, I was more focused on the activities to do, but after the training, I changed the focus to developing the documents, there are many SOPs to be developed.” (Trainee 21, NRA 10).*

Also stated were organizational adjustments primarily aimed at reducing the complexity of the regulatory activity and installing QMS without a focus on external certifications. In some settings, these efforts went along with the digitalization of regulatory activities and finetuning of the efforts towards the WHO benchmarking. Other trainees reported having established different training programs for the NRA staff and part-time dossier evaluators.

### 3.3. Areas for further assistance, improvements, and unmet expectations

**Figure 1:**
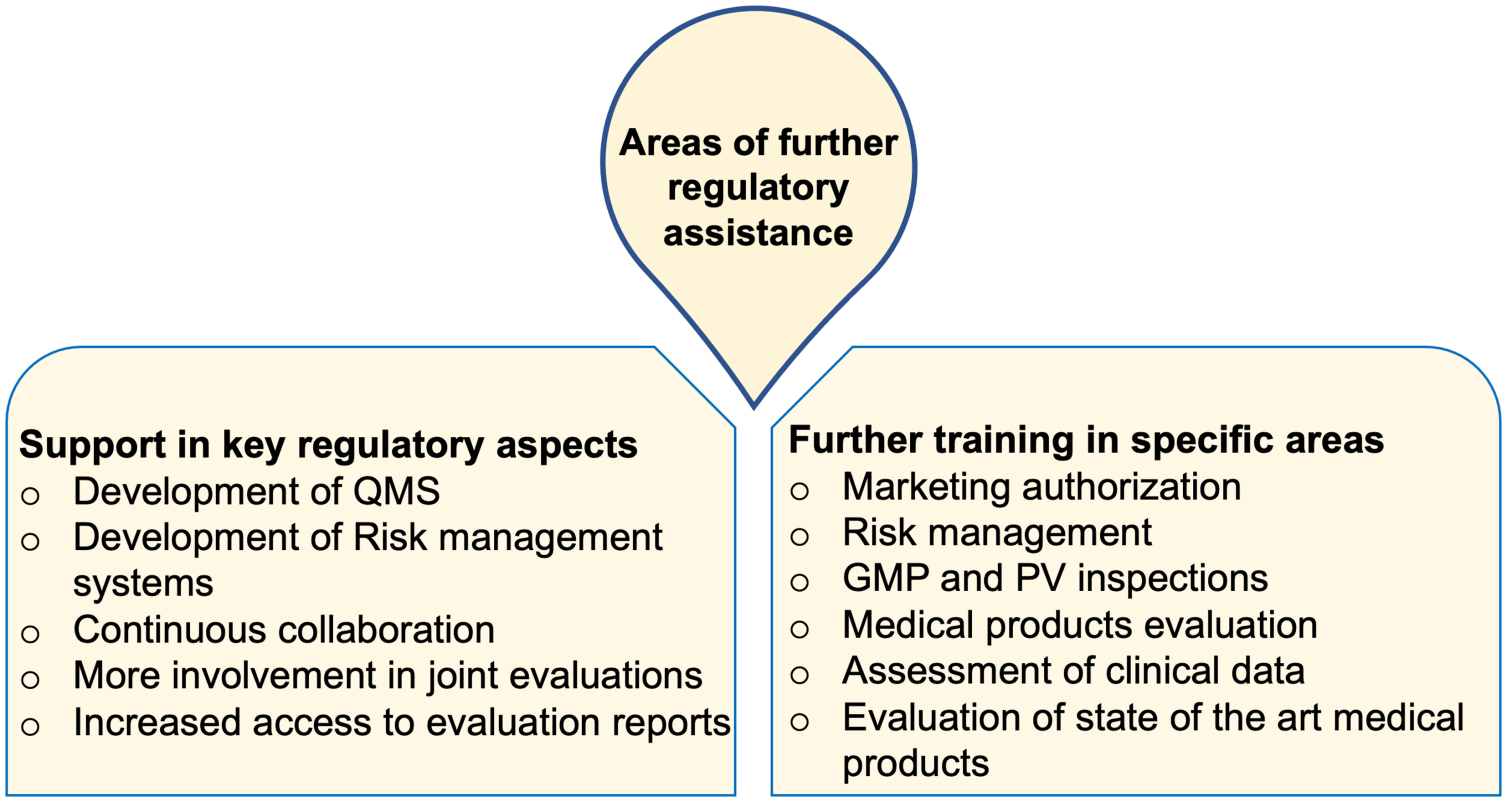
Areas of further regulatory assistance commonly stated by trainees from across the studied NRAs.

The need for further support in areas of developing functional quality as well as risk management systems, creation of continuous collaborations between NRAs, increased access to evaluation reports and training materials, and more hands-on training opportunities were highly stressed among the trainees. Through further assistance in these areas, it was hoped that the NRAs can attain more regulatory proficiency towards the provision of better and more reliable services (Figure 1).

*“There are a lot of things that we do that are not written down in any way. So, people tend to do their own things and sometimes it is by word of the mouth. Nothing is really written down anywhere except for the act and the regulations. I think that is the area that we really need to strengthen, assistance in helping us to develop a documented system before we start thinking about anything else (Trainee 17, NRA 8).*

*“… we gain more knowledge on practical regulatory training. In that way we can get better things from other regulatory authorities… we should not only be limited to lectures.” (Trainee 25, NRA 11).*

There was an agreement among many trainees that future training activities should offer more depth in the shared content. Moreover, it was proposed that more discussions should focus on the ongoing realities within the LMICs. In that, some trainees were in favour of the inclusion of more case studies and examples from the LMICs. Others suggested increasing the duration of the training while offering them more frequently.

The aspect of having hands-on training was highly emphasized and it was commonly indicated that practical exposure will add an important element to the training. Additionally, it was recommended that future training activities should aim at selecting trainees with similar levels of experience and/or inviting NRAs with similar maturity levels to each round of training. This was viewed as a way to avoid difficulties in following some of the shared contents among those with very limited levels of experience.

*“I think for me if it is possible, the training in terms of the scope should not be too wide, must be in-depth and detailed. For example, if we would like to focus on process validation, we focus on it for say 1 day and in-depth and in terms of a better understanding of process validation, rather than discussing many things just on the surface. Of course, yes you learn a lot but on the surface for a lot of things, so basically, maybe for the new officers, they may learn a lot, but for experienced officers, you just get in terms of the surface, and you might not learn a lot.” (Trainee 34, NRA 16).*

On the other hand, the Swissmedic trainers insisted on the need to find a balance between the available time and what is to be shared. They suggested more efforts be put into the preparatory phase of the training. In that, the prior sharing of guidelines and other documents to the trainees was proposed. This was postulated to allow more effective use of the available time during the training. They added that they are restricted by the necessity to observe confidentiality, which poses a big challenge in delivering hands-on training.

*“I think the confidentiality issue is really the main topic even for us. We are dealing with some information from the companies that they know that this information will not be disclosed…” (Trainer 1, Swissmedic).*

### 3.4. Regulatory preparedness during public health emergencies

The training was referred to have imparted knowledge and skills which were essential for the successful discharging of many regulatory actions under emergency situations. Some trainees reported having conducted operational threat analyses, developed relevant guidelines for Emergency Use Authorization (EUA), and put relevant technical committees in place. Other trainees mentioned the timely application of the concept of horizon scanning which enabled adequate preparations before the arrival of various Covid-19-related products. Also, through the establishment of reliance systems, and enhanced collaborations with other agencies, important regulatory actions were facilitated in the face of the Covid-19 pandemic.

*“It helped us with different procedures and steps of bringing the vaccines within the country. When we got the vaccines, it was very late and was 6 months to expire, the training really helped us on how to handle that challenge.” (Trainee 13, NRA 6).*

### 3.5. Experiences and opinions of trainers and the immediate supervisors

Trainers from the Swissmedic regarded the training as a suitable model for the provision of regulatory support. They revealed that offering the training had significantly decreased the previous workload on offering consultations to individual NRAs. Further, the trainers acknowledged receiving many questions from the trainees which could reflect the level of their interest in understanding Swissmedic’s operations. However, it was pointed out that the differences in resource availability and legal frameworks could limit the application of shared knowledge and skills.

*“I do not know to what extent the way we do things can be extrapolated to less privileged countries. But I think the way we transfer the knowledge can be a model for similar applications, where one country has the knowledge and is happy to share it with others.” (Trainer 3, Swissmedic).*

Challenges in meeting the training needs of the participants were revealed to be rooted in differences in legal frameworks and the diversity of countries participating in the training. On the other hand, the presence of an established routine and experience in offering the training, as well as the collaborative organization with the WHO-RSS team were revealed to avoid many challenges in offering the training.

*“We present the Swiss situation based on the Swiss legislation… we do not know exactly what the legal frameworks in the other countries are so that we can address our presentations for the next time in a different way… also participants in the next rounds will come from different countries, it is not so easy for us to anticipate the needs of the participants.” (Trainer 1, Swissmedic).*

The trainees’ immediate supervisors reported improved abilities in the conduction of GMP and PV inspections, more focus and in-depth assessments of the dossiers, adoption of risk-based evaluations, as well as the initiation or increased applications of reliance and horizon scanning concepts within their NRAs. Also acknowledged were more efforts among the trainees on increasing regulatory collaborations, improving QMS, planning, efficient use of resources, development of policies, and drafting of new guidelines.

Similar to the trainees, challenges related to poor organizational structures, limited number and expertise among regulatory staff, poor infrastructures and equipment, and financial constraints were also communicated by the supervisors. These challenges were stated to hinder the full realization of the benefits of the training.

### 3.6. Factors limiting effective implementation of learnt aspects

**Figure 2:**
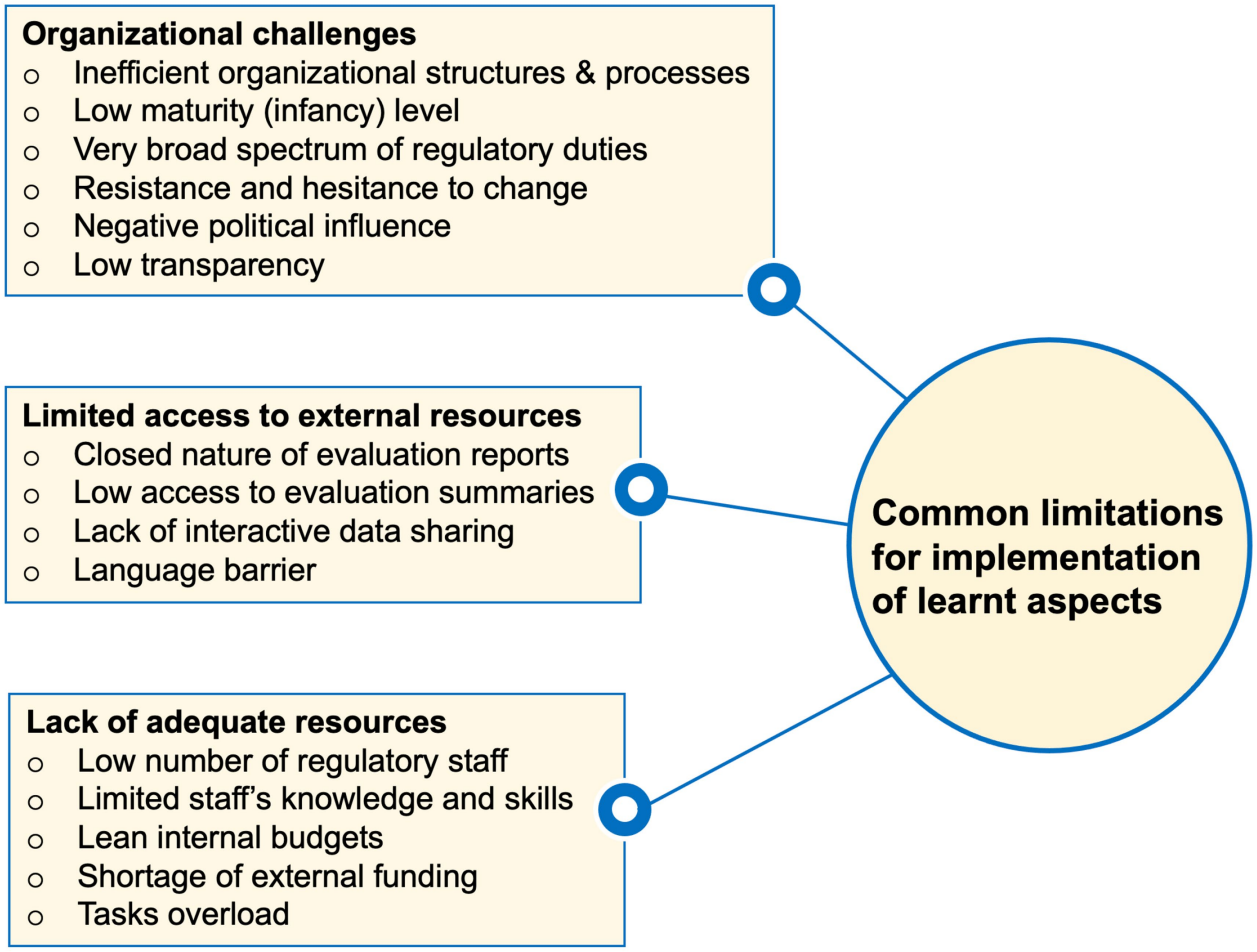
Summary of commonly highlighted limitations facing the implementation of various lessons from the WHO-Swissmedic training in trainees’ home NRAs.

Existing bottlenecks in the NRAs’ organizational structures were reported to be associated with confusing or contradicting dynamics. For example, some trainees referred to their NRAs’ involvement in the regulation of a very broad range of aspects including products, premises, and personnel to limit some of their efforts for implementing intended changes. Moreover, hesitancy to change particularly by those managing the NRAs was revealed to restrict the implementation of many learnt aspects. Similarly, limited organizational transparency, a strong negative influence of politicians, and a lack of strong legal frameworks were stated (Figure 2).

*“The difficulty is that a lot of time you have to convince the management and the executive on the benefits of adopting some of these approaches… the challenge is to get the management to buy your proposal for changes to be implemented. It is a complicated situation…” (Trainee 14, NRA 7).*

Furthermore, lacking direct access to evaluation data summaries, the closed nature of evaluation reports, and language barriers were indicated to limit the evaluation of products based on reliance. Other trainees implicated the lack of adequate knowledge and skills, understaffing, high turnover rates among the experienced staff, limited internal budgets and shortage of external funding as aspects impairing their efforts to implement the learnt aspects (Figure 2).

*“At the moment we are only two regulators here, headed by the chief pharmacist, who also looks after other units, so it is not only us. The regulatory work is done by just the two of us.” (Trainee 30, NRA 14).*

In addition to better experiences reported in implementing learnt aspects among the WHO Maturity Level 3 (ML3) NRAs, the nature of the prevailing limitations was noted to differ depending on the maturity level. For example, while participants from the NRAs with lower maturity levels reported inadequate primary infrastructures, a lack of QMS and functional legal framework, those from the ML3 NRAs highlighted challenges related to further training of the available staff, installing measures to regulate novel medical products, establishing functional IT-based systems, a few to mention.

## 4. Discussion

### 4.1. Approaches in trainees’ selection and meeting their expectations

This study has revealed different approaches for the selection of NRA staff to participate in training opportunities. Among other aspects, more priority should be given to those which grant training opportunities to the staff with the highest training needs. Only about 14% of regulatory professionals were previously reported to have primary training related to regulatory work, indicating a huge on-job training need (5). While the selection of staff in higher managerial positions is common, there is a huge need for training opportunities specifically addressing leadership aspects in regulatory affairs (4, 5, 7). Furthermore, the unmet training expectations among some trainees could be addressed *via* regular training needs analyses, among other aspects, as discussed further in the subsequent sections (1, 5).

### 4.2. Key lessons and their implementation

Among other highlighted lessons from the training, an increased understanding of the concept of reliance in regulatory activities is crucial in fighting the wrong notions that matured NRAs are self-sufficient or that relying on others signifies weakness. In that respect, recent findings have shown the growing use of reliance across many regulatory agencies (12, 13). Moreover, lessons on having functional regulatory systems encourage the shift in regulators’ focus towards the creation and strengthening of suitable systems for increased regulatory proficiency. This shift should however go in hand with the favourable allocation of the available resources towards having effective structures and systems for the competent execution of core regulatory functions, instead of prioritizing external accreditations (14, 15).

Furthermore, since NRAs in the LMICs are mostly faced with limited availability of resources, capitalizing on the lessons around careful planning, measures to shorten various timelines, outsourcing of expertise, and the use of Information Management Systems (IMS) could minimize the associated challenges (4, 15, 16). This is exemplified by the reported improvements in regulatory efficiency and collaborations among the East African Community (EAC) partner following the implementation of IMS within their regulatory harmonization initiative (17).

The reported implementation of various learnt aspects by the trainees underscores the role of regulatory training in the strengthening of regulatory systems in the LMICs. In that, the NRAs in the LMICs can potentially attain more robust and thorough regulatory processes towards better access to safe, effective, and good-quality medical products (12, 13). Moreover, the shared success stories from the trainees signify the possibility of implementing sustainable changes in the whole regulatory landscape and in turn gaining more confidence and satisfaction from stakeholders, customers, and the public (14, 18–20). While most of the NRAs in the LMICs are faced with high workloads among the few available staff, taken together, the implementation of the learnt aspects could address this challenge and enable proper allocation of human and other resources (21, 22).

### 4.3. Limitations of effective implementation of learnt aspects and possible mitigations

The full realization of the benefits of regulatory training requires effective measures to address the various challenges affecting the implementation of learnt aspects highlighted in this study. These should include streamlining the nature and scope of discharged regulatory functions, adopting more effective organizational structures, (16, 17, 20, 21, 23, 24) and increasing willingness to undertake and sustain organizational changes (15, 25, 26).

Moreover, the need for observing confidentiality was painted at the centre of the difficulties in accessing product evaluation reports from SRAs. This and related aspects like the lack of mutual trust, and differences in regulatory and legal frameworks potentially impair the complete realization of the benefits of implementing the concept of reliance (12, 17, 23, 27). Furthermore, the challenge of shortages in resources in many of the NRAs in the LMICs is deeply embedded within low national budgets and the presence of other priority areas at the national level (15, 28). Sustainable mitigation of this challenge, therefore, requires an active search for external funding opportunities, as well as employing various collaborative approaches in the discharge of regulatory functions (15, 28).

### 4.4. Areas of further assistance to increase access to regulatory training opportunities

The high demand for further regulatory assistance revealed in this study could be explained by the dramatic changes in modern regulatory sciences driven by rapid technological advances in drug discovery and development. Recent findings have reported the presence of minimal or complete lack of capacity to execute medicines regulation functions in more than 90% of the NRAs in Africa (6). Moreover, an increased need for the establishment of more structured and autonomous regulatory agencies around the world created higher demands for regulatory support (6, 29). Further, based on the constantly and rapidly evolving regulatory landscape, the need for continuous improvements of regulatory systems is crucial. Ultimately, this calls for more coordinated efforts by the WHO and other well-wishing organizations to carry forward the heavy but noble duty of providing regulatory support (16, 22, 30, 31).

The existing high need for various sorts of regulatory training is related to the very low availability of advanced formal training in health products regulatory affairs within the LMICs (5). Additionally, the common presence of generalized curricula lends low priority to regulatory aspects and offers limited practical exposure (5, 6, 7). This suggests the need for alternative ways to ensure the acquisition of practical skills without breaches of confidentiality. Among the possible solutions are the use of dummy dossiers and the creation of dedicated databases for sharing anonymous data/case studies. The experience from current training opens a door for possible recruitment of other parties in joining the WHO and Swissmedic’s efforts towards the organization, provision of trainers, training facilities and materials, as well as finances and other resources towards the enriching, expanding, and sustaining the ongoing training efforts (3, 32, 33).

The current study has indicated that the execution of many regulatory functions during the Covid-19 pandemic was facilitated by the application of knowledge, skills and principles gathered from the training, particularly the concepts of recognition and reliance, as well as horizon scanning. These findings were similar to the recent reports on the presence of big changes in regulatory practices brought about by the Covid-19 pandemic and necessitate detailed training on regulatory preparedness in public health emergencies (12, 13, 34, 35).

The execution of proposed improvements on the training is however dependent on the core objectives and scope for its establishment and the implicated additional resources. Nevertheless, revising the objectives for the possible accommodation of the given suggestions is highly recommended. In general, the need for more resources to implement such suggestions calls for the possibility of other agencies to join these valuable efforts in offering support in all or selected aspects (15, 29, 37).

Improved capabilities of the trainees in discharging their regulatory roles were acclaimed by their immediate supervisors to have advanced the NRAs’ performances on different frontiers. On the other hand, the suitability of the training in the provision of regulatory support to NRAs in LMICs was echoed by the trainers. However, based on the increased gradual increased interest in the training, continuous improvements to the program are needed (4, 5, 7, 33). Among other aspects, measures to expand the trainers’ base, involving trainers from LMIC settings, and inviting other NRAs from similar settings to assume some of the available roles in offering similar training are warranted (7, 32, 33).

### 4.5. Recommendations

Considering the highlighted huge need for further regulatory support among the NRAs in the LMICs, further broad and well-coordinated efforts in improving the current training and offering other similar opportunities are warranted. Thus, the following set of measures is highly recommended:

- Ensuring offering of regular training opportunities to regulatory staff based on both individual and organizational capacity-building needs.
- NRAs in LMICs should ensure proper planning and allocation of the available resources towards enabling the effective execution of their core regulatory functions.
- To maximize the available benefits, internal challenges limiting the effective implementation of lessons learnt from different training activities should be continuously addressed.
- In addition to regulatory training, the WHO and other interested parties should continue strengthening other sustainable and inclusive means for the provision of regulatory support.
- Future training activities should seek to include trainers, case studies, and experiences from NRAs based in the LMICs.
- Other NRAs and related institutions capable of offering any form and scope of regulatory training should highly consider doing so.
- With the experience and lessons from the ongoing training activities, the WHO should seek to address the existing challenges in organizing future training activities.

### 4.6. Study limitations and strengths

Although all the interviews were conducted by a single interviewer who had no previous link with the training project, two of the researchers involved in the data coding and analysis were involved in the overall organization of the training activities. Thus, the possibility of compromised independence of the coding and analysis cannot be entirely excluded. Moreover, the qualitative nature of this study hindered the benchmarking of the level of participants’ knowledge and other quantitative indicators before the training and their follow-up in later stages, so as to generate more evidence on the overall impact of the training.

Besides, the strengths of this study include its qualitative nature which allowed for the in-depth exploration of the aspects related to lessons learnt, practices, and experiences among the trainees, their immediate supervisors as well as the trainers. In that, the trainees involved in this study had different levels of expertise in areas representative of all core regulatory functions. Moreover, the involvement of those groups favoured the evaluation of the underlying constructs from different perspectives. Also, this study has presented the findings representative of the views of trainees from NRAs having a broad diversity in their geographical locations, economic statuses and levels of regulatory maturity.

## 5. Conclusion

Training regulators as a means of providing regulatory support is a promising approach to promoting sustainable advancements in the regulatory landscape among the LMICs. The potential hosted in this modality is nevertheless hindered by the limited availability of training opportunities. In addition to the need for suitable trainers, of essence are also coordinated identification of training needs, financing, and the overall organization of the training activities.

The current study has revealed high relevance and key contributions of the ongoing collaborative training of the regulatory workforce from the NRAs located in the LMICs across the world. Many learnt aspects across all key domains of regulatory practices were shared by the trainees. In addition, the existence of widespread efforts to ensure their successful implementations in local settings was implicated by the trainees and mirrored by their supervisors. Moreover, the training activities were reflected to offer much-needed support to meet an array of knowledge, skills and resources required to enable effective regulatory responses in times of public health emergencies.

However, several challenges rooted in the organizational structures and process, availability of resources as well as geographical and political diversities among others, were revealed to limit the optimal realization of the benefits of the training.

## Data availability statement

The original contributions presented in the study are included in the article/Supplementary material, further inquiries can be directed to the corresponding author.

## Ethics statement

Ethical review and approval was not required for the study on human participants in accordance with the local legislation and institutional requirements. Written informed consent to participate in this study was provided by the participants. Written informed consent was obtained from the individuals for the publication of any potentially identifiable images or data included in this article.

## Author contributions

AK: conceptualization, study design, data collection, data analysis and interpretation, visualization, validation, writing the original draft, review, and editing. RD and LP: conceptualization, study design, literature search, data collection, data analysis and interpretation, visualization, validation, writing the original draft, review, and editing. HS: conceptualization, study design, validation, writing the original draft, review, and editing. All authors contributed to the article and approved the submitted version.

## Funding

This study was funded by the World Health Organization.

## Conflict of interest

RD, AK, and HS are working with the World Health Organization. LP is working with the Swissmedic.

## Publisher’s note

All claims expressed in this article are solely those of the authors and do not necessarily represent those of their affiliated organizations, or those of the publisher, the editors and the reviewers. Any product that may be evaluated in this article, or claim that may be made by its manufacturer, is not guaranteed or endorsed by the publisher.
