# Supplementary material for: Collaborative training of regulators as an approach for strengthening regulatory systems in LMICs: experiences of the WHO and Swissmedic
Source: Front Med (Lausanne). 2023 May 18;10:1173291. doi: 10.3389/fmed.2023.1173291 (PMC10233123; doi:10.3389/fmed.2023.1173291)
Supplement: Supplementary file 1 [file Data_Sheet_1.PDF]

In-depth Interview guide for the Swissmedic trainers

A. When all expected participants have joined the meeting:

i. *Welcome all participants to the meeting and apologize in case the meeting has not started on the expected time due to e.g., waiting for other participants or technological difficulties.*
ii. *Introduce yourself: Full name, your professional background, and your role in this study*
iii. *Ask for each participant to introduce him-/herself: Name, professional background, and current role in the NRA.*
iv. *Thank the participants for the introduction and re-introduce the study and its main objectives.*
v. *Remind the participants on the way the interview will be conducted, state your role as an independent party, re-assure them on confidentiality of their identities, as well as their right to withdraw from the study at any time.*
vi. *Ask if there is any question or need for further clarifications. If available take note and answer accordingly.*

B. Proceed with the interview by asking the following questions audibly and articulately.

Please observe the following during the interview:

i. *Depending on the flow and state of the responses during the interview, you may skip to the most appropriate next question(s) and return to other question(s) later.*
ii. *Ensure a regular change in the order in which the participants are to respond to the questions.*
iii. *Allow the participants to give additional responses in case they remember something at the later stage.*
iv. *Ensure that each of the available participant has been given an opportunity to respond to each question.*

Questions/probes

Part I

i. To what extent do you think the WHO-Swissmedic training activities are reaching their objectives?

ii. How are you satisfied with the participants' engagement and participation during the training?

iii. How have the training helped you to identify different challenges faced by regulators from different areas around the world?

iv. To what extent do you think the training is standing as a model for provision of regulatory support.

v. What are the major challenges which you face in offering the training?

Part II

Explain to the interviewees that this part contains questions which are rooted from the experiences/responses shared by the trainees.

i. What is your say on the suggestion that the training should involve more interactions between trainers and them and to include more hands-on sessions?

ii. The participants wish that the training should offer more depth, more detailed information from trainers, and more specific issues, examples and case studies. To what extent is this doable?

iii. Participants feel like time for the training was not enough in comparison to the of materials that are to be covered, there is a feeling of a rush to complete the syllabus or finish the contents to be delivered. What is your opinion on this aspect?

iv. How do you think the selection of participants based on their knowledge and experience in regulatory activities will be helpful to the overall training programme.

v. In your view, how suitable is the approach to involve NRAs with similar maturity levels only in each particular round of the training?

vi. Some trainees see a need for a continuous support in some learnt aspects, however but they shared on the difficulty in accessing the trainers for follow up questions or discussions after the training. In that, they wished for an online platform for a continuous sharing of regulatory information. What are your views on this proposal?

vii. The trainees would prefer to have on-site training to the online/virtual training modality. What are your preferences as a trainer? Are there any reasons?

viii. How ready are you to travel to another country to offer a 2-week long training to trainees from a number NRAs in that particular region?

C. After the interview section is completed:

a. *Thank all the participants for their participation to the interview.*
b. *Highlight the possibility of contacting them in the future in case of the need for additional information.*
c. *Ask for any further questions from the participants, if available answer them accordingly*
d. *Once again thank the participants and end the meeting for all.*
