# Supplementary material for: Collaborative training of regulators as an approach for strengthening regulatory systems in LMICs: experiences of the WHO and Swissmedic
Source: Front Med (Lausanne). 2023 May 18;10:1173291. doi: 10.3389/fmed.2023.1173291 (PMC10233123; doi:10.3389/fmed.2023.1173291)
Supplement: Supplementary file 2 [file Data_Sheet_2.PDF]

Guide for in-depth interviews with trainees from NRAs

A. When all expected participants have joined the meeting:

i. *Welcome all participants to the meeting and apologize in case the meeting has not started on the expected time due to e.g., waiting for other participants or technological difficulties.*
ii. *Introduce yourself: Full name, your professional background, and your role in this study*
iii. *Ask for each participant to introduce him-/herself: Name, professional background, and current role in the NRA.*
iv. *Thank the participants for the introduction and re-introduce the study and its main objectives.*
v. *Remind the participants on the way the interview will be conducted, state your role as an independent party, re-assure them on confidentiality of their identities, as well as their right to withdraw from the study at any time.*
vi. *Ask if there is any question or need for further clarifications. If available take note and answer accordingly.*

B. Proceed with the interview by asking the following questions audibly and articulately.

Please observe the following during the interview:

i. *Depending on the flow and state of the responses during the interview, you may skip to the most appropriate next question(s) and return to other question(s) later.*
ii. *Ensure a regular change in the order in which the participants are to respond to the questions.*
iii. *Allow the participants to give additional responses in case they remember something at the later stage.*
iv. *Ensure that each of the available participant has been given an opportunity to respond to each question.*

Questions/probes

i. Could you please describe the circumstances under which you were nominated to take part in the WHO-Swissmedic training? Why did you decide to take part in this training?
ii. What were the main things which you learnt during the training?
iii. How have you been applying the knowledge and skills obtained from the training? (What are you doing differently compared to before the training?).

iv. Have there been any challenges or limitations in implementing the obtained knowledge or skills within your NRA? Would you like to share them with me/us?
v. Is there a need for further assistance from outside your NRA in implementing the lessons from the training? Please highlight the areas in which you think further assistance is needed.
vi. In what ways have the training activities enhanced your competence in regulatory preparedness during public health emergencies, taking an example of Covid-19?
vii. How was the training useful creating opportunities for meeting and exchange of experiences with regulatory experts from other countries?
viii. Have you managed to share the obtained knowledge and skills to others within your NRA? In what ways have this been done/achieved?
ix. What are topics/areas of work that you considered to be important but were not addressed in training?
x. What would you recommend on any aspect of the training?

C. After the interview section is completed:

a. *Thank all the participants for their participation to the interview.*
b. *Highlight the possibility of contacting them in the future in case of the need for additional information.*
c. *Ask for any further questions from the participants, if available answer them accordingly*
d. *Once again thank the participants and end the meeting for all.*
