# Supplementary material for: Collaborative training of regulators as an approach for strengthening regulatory systems in LMICs: experiences of the WHO and Swissmedic
Source: Front Med (Lausanne). 2023 May 18;10:1173291. doi: 10.3389/fmed.2023.1173291 (PMC10233123; doi:10.3389/fmed.2023.1173291)
Supplement: Supplementary file 3 [file Data_Sheet_3.PDF]

Follow-up Questionnaire to immediate supervisors of the previous participants in the WHO-Swissmedic Collaborative training of Regulators.

(You can type-in your responses directly on this soft copy OR you can print it and fill in by hand)

Name and country of the Regulatory Authority	
Name of the immediate supervisor	
Name(s) of the supervisee(s) here referred to	
Name of the Unit/ Department/ Division/ Agency under your supervision (please cross the unwanted)	

Qn. 1	In what ways do you see that the WHO-Swissmedic training has have helped your supervisee's ability to carry out the regulatory duties under your supervision?

Qn. 2	What are the factors limiting the implementation of the knowledge and skills acquired from the WHO-Swissmedic training in the settings/area under your supervision?
Qn. 3	Do you have any other comments regarding the WHO-Swissmedic training?

Please send the scanned copies of the <u>signed consent form</u> and the <u>filled questionnaire</u> to the email address:
